# Glucocorticoid receptor activation reorganizes Wnt/LEF1 regulatory circuitry associated with therapeutic response in B-cell acute lymphoblastic leukemia

**DOI:** 10.64898/2026.09.23.752002

**Published:** 2026-09-24

**Authors:** Robert J. Mobley, Damilola Oluwalana, Gabriele Baniulyte, Minzhang Zheng, Brett Brown, Faten Usrof, Kashi Raj Bhattarai, Kelly R. Barnett, Virginia Valentine, Marc Valentine, Seth Staller, Satoshi Yoshimura, Kristine R. Crews, David T. Teachey, Steven Burden, Yong Cheng, Jun J. Yang, Daniel Savic

## Abstract

Glucocorticoids (GCs) are foundational to B-cell acute lymphoblastic leukemia (B-ALL) therapy, yet how glucocorticoid receptor (GR) activation interacts with lineage regulatory circuitry to shape therapeutic response remains incompletely understood. Using LEF1 to interrogate canonical Wnt regulatory circuitry, we show that GR activation reorganizes GR+LEF1 elements into recruitment, retention, and dissociation states. LEF1 dissociates from Wnt-responsive regulatory elements while being recruited to GRE-rich GR-associated elements that progressively acquire chromatin accessibility, H3K27ac, BRD4 and BRG1. RUNX1 and AP-1 show class-dependent redistribution across LEF1-defined states, extending organization beyond LEF1. GR+LEF1 reorganization is observed in patient-derived B-ALL. GR+LEF1 elements lose accessibility in GC-resistant B-ALL and during diagnosis-to-relapse evolution, with recruitment elements enriched for remodeling. Perturbation of resistance-associated GR+LEF1 elements at BIM and BMF alters GC-induced apoptotic signaling. Together, these findings identify Wnt/LEF1 circuitry as an interface through which therapeutic GR activation reorganizes the B-ALL regulatory landscape into states associated with glucocorticoid response and resistance.

## Full Text Availability

The license terms selected by the author(s) for this preprint version do not permit archiving in PMC. The full text is available from the preprint server.

